# Isolation and Whole Genome Sequence Analysis of *Alcaligenes* and *Chromobacterium* Strains with Antimicrobial Activity Against ESKAPE Pathogen Relatives

**DOI:** 10.7150/jgen.115133

**Published:** 2025-06-23

**Authors:** Hannah W. Lwin, Jonathan D. Dattelbaum

**Affiliations:** 138 UR Drive, Department of Chemistry, University of Richmond, Richmond, VA 23173, USA.

**Keywords:** ESKAPE pathogens, antimicrobial, antibiotic resistance

## Abstract

Discovery, development, and production of new antibiotic drugs in a form safe for human consumption have become increasingly difficult, expensive, and time-consuming, especially with an increase in antibiotic-resistant pathogens. The ESKAPE pathogens are a group of six pathogenic bacteria that can be highly virulent and are likely to, or already have, developed antibiotic resistance to many of the currently available antibiotics. New antibiotics or new activities of existing natural products are needed to combat these multi-drug resistant pathogens. Our approach was to search for soil microbes that produce antimicrobial compounds that could potentially inhibit the growth of the ESKAPE pathogens. We report one draft genome of *Chromobacterium* and one draft genome of *Alcaligenes* cultured from soil with antimicrobial activity against *Staphylococcus epidermidis*, a relative of ESKAPE pathogen *Staphylococcus aureus*. The lengths of the genomes were 5.2 and 4.0 Mbps and GC content was at 64.4% and 56.1% for *Chromobacterium sp.* HL1 and *Alcaligenes parafaecalis* HL2, respectively. *Chromobacterium sp.* HL1 has not been assigned to any previously known species. Phylogenetic analysis revealed that *Chromobacterium sp.* HL1 may be closely related to *Chromobacterium fluminis* and *Chromobacterium alkanivorans*. *A. parafaecalis* HL2 is likely related to *Alcaligenes faecalis subsp. parafaecalis*. Functional analysis revealed biosynthetic gene clusters related to betalactone, terpene, isocyanide, and T1PKS in one or both genomes analyzed. Antimicrobial properties were previously reported from the products of these gene clusters that could further aid our search for the active component of the analyzed strains.

## Introduction

ESKAPE is an acronym refering to six pathogenic organisms that are considered to be highly virulent and are likely to, or already have, developed antibiotic resistance to many of the currently available antibiotics. They are *Enterococcus faecium*, *Staphylococcus aureus*, *Klebsiella pneumoniae*, *Acinetobacter baumannii*, *Pseudomonas aeruginosa*, and several *Enterobacter* species. These organisms are involved in the majority of antibiotic-resistant infections in healthcare settings 1, and thus it is critical to find new antibiotics or new activities of existing natural products to combat these multi-drug resistant pathogens. Soil environments contain an immense diversity of inhabiting microorganisms, including bacteria that naturally produce antimicrobial compounds 2. Therefore, our approach was to search for soil microbes that produce antimicrobial compounds that could potentially inhibit the growth of the ESKAPE pathogens.

*S. aureus* is a gram-positive bacteria and is the cause of millions of severe, invasive infections worldwide 3. In the US alone, nearly 120,000 *S. aureus* infections and 20,000 associated deaths occurred in 2017 4. Treatment is difficult due to multi-drug resistant *S. aureus* strains, such as Methicillin-sensitive *S. aureus* (MSSA) and Methicillin-resistant *S. aureus* (MRSA) that are prevalent in medical settings 5. There are few antibiotics left that can treat MRSA infections, such as vancomycin; moreover, these multi-drug resistant strains can develop resistance easily, as seen with some vancomycin-resistant strains that have appeared already 3,6. Thus, new potential medications are needed to combat these strains while a long term solution is determined for the increasing antibiotic resistance of pathogens overall.

*Alcaligenes* is a genus of gram-negative bacteria, including *Alcaligenes faecalis*, *Alcaligenes nematophilus*, and a few other species, isolated from soil and aquatic environments. *A. faecalis* in particular may produce tunicamycin, an antimicrobial agent that may have potential for antibiotic development 7. On the other hand, *Chromobacterium* is a genus of gram-negative, rod-shaped bacteria, including *Chromobacterium violaceum*, *Chromobacterium rhizoryzae*, and a few other species, isolated from soil and aquatic environments. *Chromobacterium violaceum* in particular is known to produce violacein, a violet pigment with potential antibiotic properties, among many other functions 8. There is limited research regarding other species within the *Alcaligenes* and *Chromobacterium* genera about their secondary metabolites, and thus there may be undiscovered antimicrobial compounds that could potentially be used for new antibiotics.

This study explores the characteristics of two genomes of bacteria, isolated from soil sources located in Richmond, VA in the United States. One genome sequence of *Chromobacterium sp.* and one genome sequence of *Alcaligenes parafaecalis* are reported.

## Materials and Methods

One strain of *Chromobacterium sp.* and one strain of *Alcaligenes parafaecalis* were isolated from soil in Richmond, Virginia. *Chromobacterium sp.* was isolated from soil near Abby's garden in the Eco-Corridor on the University of Richmond campus, and *A. parafaecalis* was isolated from compost. Strains were cultured at room temperature (~21^o^C) on plates containing Luria Broth (LB) media with 10 µg/mL cycloheximide to inhibit fungal growth. Medium was solidified with 1.5% agar. Strains were assayed against safe ESKAPE pathogen relatives for antimicrobial activity, namely *Staphylococcus epidermidis*. For genomic DNA extraction, Zymo Quick-DNA Fungal/Bacteria Miniprep Kit (Zymo Research) was used according to the manufacturer's protocol.

Whole genome sequencing was conducted by Plasmidsaurus (San Francisco, CA). Amplification-free long-read sequencing libraries were prepared using the v14 library prep chemistry (Oxford Nanopore Technologies (ONT)).The libraries were sequenced with a primer-free protocol using R10.4.1 flow cells (ONT). The bottom 5% worst fastq reads were removed via Filtlong v0.2.1 on default parameters 9. Furthermore, the reads were downsampled to 250 Mb via Filtlong to create a rough sketch of the assembly with Miniasm v0.3 10. The reads were re-downsampled to ~100x coverage, or nothing if there was not at least 100x coverage, with heavy weight applied to remove low quality reads. A Flye v2.9.1 assembly was run with parameters selected for high quality ONT reads 11. The Flye assembly was polished via Medaka v1.8.0 12 using the re-downsampled reads. Contig analysis was performed using Bandage v0.8.1 13. Contamination and completeness of assemblies were calculated using CheckM v1.2.2 14. Assemblies were polished with Illumina .fastq reads using Polypolish v0.6.0 15. Genome annotation was performed using Bakta v1.6.1 16 and the eggNOG-mapper website v5.0.0 17. Biosynthetic gene clusters (BGCs) prediction was conducted with antiSMASH v7.1.0 18. Taxonomic annotation of genomes was performed with Mash v2.3 19 against RefSeq genomes and plasmids and Sourmash v4.6.1 20 against GenBank. Phylogenomic analysis was accomplished on Type (Strain) Genome Server (TYGS) 21, and the whole-genome and 16S rRNA gene sequence-based phylogenetic trees were visualized using iTOL version 1.0 22. For TYGS analysis, 28 reference strain genomes were used. They are available in the NCBI database under accessions: GCA_021129175, GCA_021129195, GCA_016937655, GCA_011602385, GCA_002902845, GCA_002924365, GCA_003693445, GCA_008275125, GCA_000711885, GCA_001855555, GCA_001676875, GCA_001953795, GCA_000007705, GCA_000971335, GCA_023913775, GCA_002837135, GCA_019343455, GCA_000429385, GCA_003545825, GCA_000739855, GCA_014652815, GCA_014635265, GCA_011927625, GCA_026344155, GCA_902859645, GCA_026344135, GCA_026344035, GCA_022230885.

All sequencing data are publicly available from the National Institutes of Health under BioProject accession PRJNA1134774 and GenBank accession CP161982 for *A. parafaecalis HL2*, and BioProject accession PRJNA1134732 and GenBank accession CP162399 for *Chromobacterium sp*. *HL1*.

## Results and Discussion

*Alcaligenes parafaecalis* HL2 and *Chromobacterium sp.* HL1 were both observed to have antimicrobial activity against *Staphylococcus epidermidis*, a relative of ESKAPE pathogen *S. aureus*. This suggests that both strains may produce a compound(s) that could have activity against *S. aureus*. In order to determine the active component of both strains' observed antimicrobial activity, their genomes were sequenced and annotated.

Both draft genomes were composed of 1 contig, with genome sizes 4.0 and 5.2 Mbps for *A. parafaecalis* HL2 and *Chromobacterium sp.* HL1, respectively. The overall genome completeness was estimated at 100% and 99.15%, with contamination at 0% and 0.85% and GC content at 56.1% and 64.4%. The summary is presented in (Table 1).

**Figure 1:**
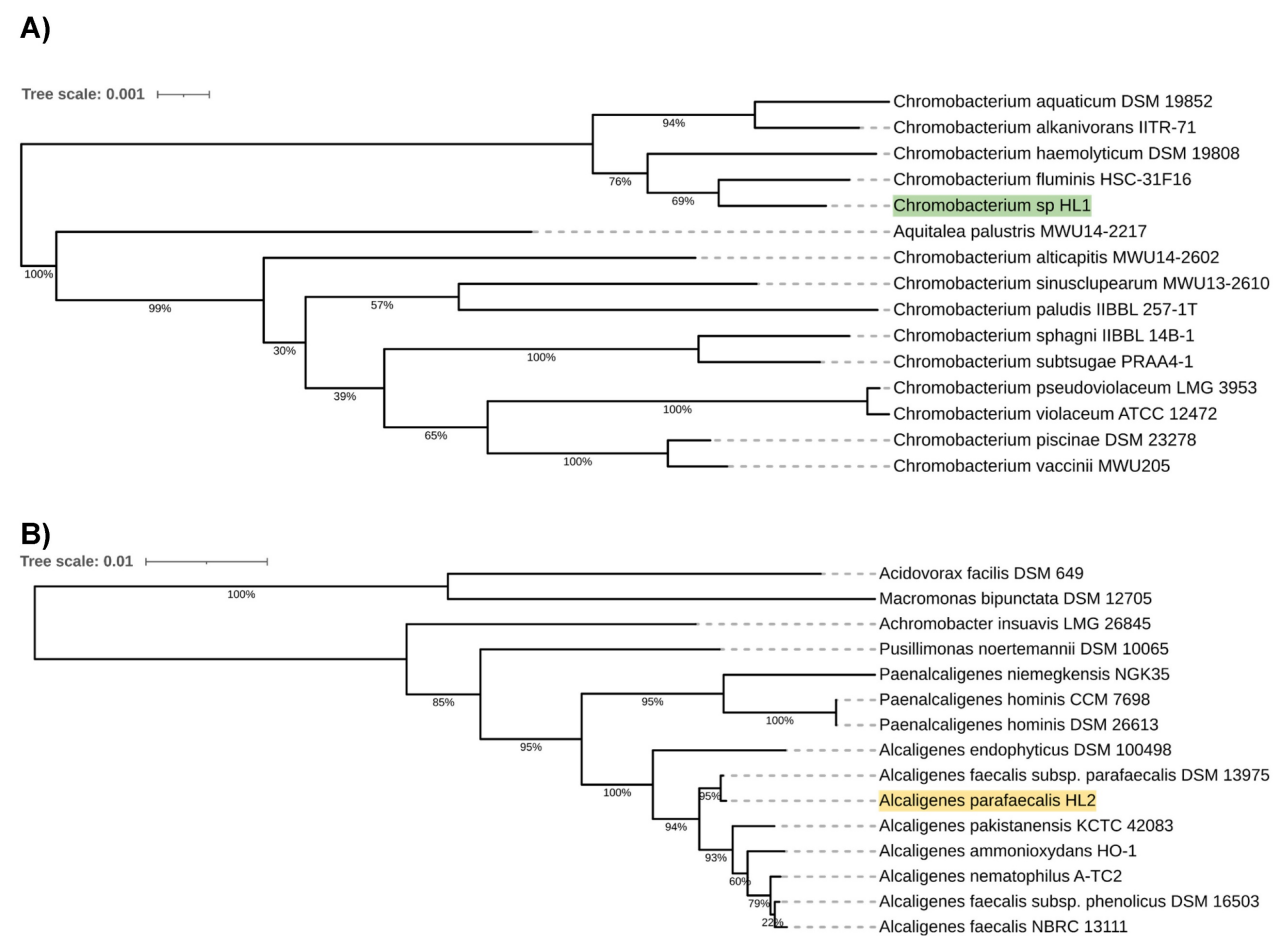
** A.** *Chromobacterium* sp. HL1 16S rRNA sequence-based phylogenetic tree. **B.** *Alcaligenes parafaecalis* HL2 16S rRNA sequence-based phylogenetic tree.

Preliminary taxonomic annotation of genomes using Mash and Sourmash assigned isolate HL1 to *Chromobacterium* genus and isolate HL2 to *Alcaligenes* genus. HL2 was further assigned to *A. parafaecalis* species. The assignment to the species level couldn't be determined for HL1 because of the high degree of difference in genome sequences between the analyzed isolate and the previously described genomes available in the databases, suggesting a potential new species of *Chromobacterium*. A phylogenetic analysis was performed in order to deepen knowledge about the relationship between the analyzed isolates and other species. Based on the 16S rRNA gene sequences, the resulting phylogenetic tree confirmed that isolate HL1 fell within a group comprising members of the genus *Chromobacterium* and that isolate HL2 fell within a group including members of the genus *Alcaligenes* (Figure 1A, 1B).

**Figure 2:**
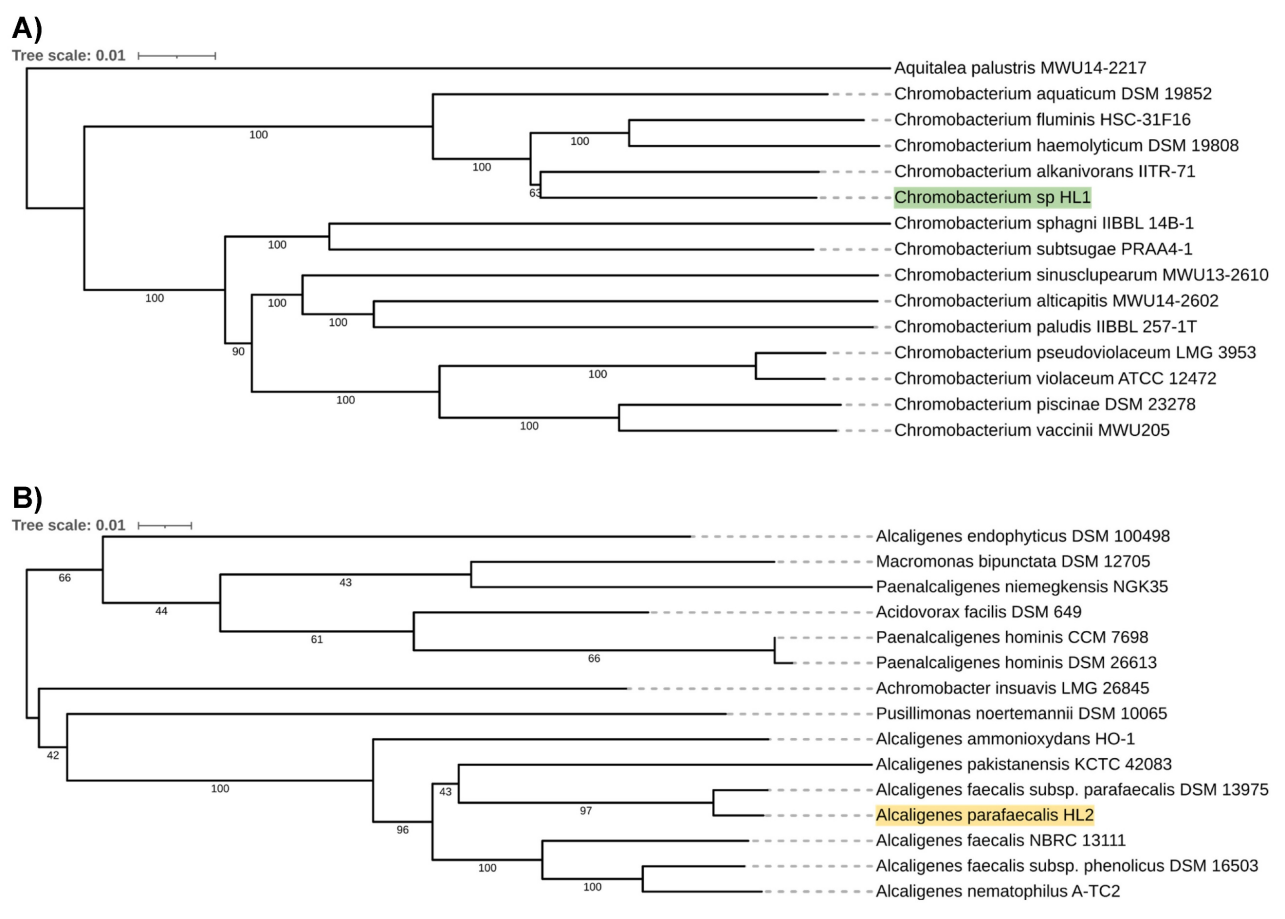
** A.** *Chromobacterium sp.* HL1 genome sequence-based phylogenetic tree. **B.** *Alcaligenes parafaecalis* HL2 genome sequence-based phylogenetic tree.

However, in both cases, the analyzed isolates were separated from the other species included in the analysis. The closest species for *Chromobacterium sp.* HL1 was *Chromobacterium fluminis*. In the case of *A. parafaecalis* HL2, the closest taxon was *Alcaligenes faecalis subsp. parafaecalis*. This initial phylogenetic analysis, using a comparison of 16S rRNA gene sequences, was then deepened through the construction of a further, whole-genome sequence-based phylogenetic tree built using Genome BLAST Distance Phylogeny approach (GBDP) created on the TYGS platform (Figure 2A, 2B). On the genome-wide scale, it was noticed that *Chromobacterium sp.* HL1 was more distant from *Chromobacterium fluminis* and that the closest species was *Chromobacterium alkanivorans*. That may suggest genomes assignment to two different species of *Chromobacterium* and possibly a new species of *Chromobacterium*. As for *A. parafaecalis* HL2, the closest related species was *A. faecalis subsp. parafaecalis*, similar to the 16S rRNA phylogenetic analysis.

Functional annotations revealed that both draft genomes contained numerous genes involved in the biosynthesis of secondary metabolites (Table 2). Moreover, *A. parafaecalis* HL2 had a higher number of genes belonging to this category (102 genes) than *Chromobacterium sp.* HL1 (91 genes). To further explore these secondary metabolites biosynthesis genes, the annotation of BGCs with antiSMASH was performed. Interestingly, more BGCs were identified in the *Chromobacterium* strain than *Alcaligenes*, despite a smaller number of genes associated with processes identified during the analysis. Moreover, BGCs related to betalactone, hydrogen-cyanide, and terpene production have been identified in both genomes. In *Chromobacterium sp.* HL1, BGCs associated with homoserine lactone and isocyanide production were also identified in its genome. In *A. parafaecalis* HL2, BGCs related to the production of ectoine and Type I polyketide synthase (T1PKS) were also identified. Table 3 summarizes the information on the identified BGCs in each of the strains.

Betalactone, terpene, isocyanide, and T1PKS were previously reported to produce small molecules with antimicrobial properties 23-26. This gives some insight into what may be the active component of both strains' observed antimicrobial activity against *S. epidermidis*. Currently, work is being done on isolating the active component from liquid cultures of varying growing conditions via column chromatography, methanol extraction, and rotary evaporation to potentially characterize the active component. Determining the gene cluster responsible for the active component would help speed up the process in identifying the antimicrobial compound(s) and determining its effectiveness against *S. aureus*.

In summary, two draft genomes of one *A. parafaecalis* strain and one *Chromobacterium sp.* strain expands the genomic knowledge of each genera. The prevalence of BGCs, that produce antimicrobial compounds, in both genomes provide a potential lead in identifying the active component of both strains against *S. epidermidis*. Further research will attempt to identify the active component and determine its effectiveness against *S. aureus* and ultimately may serve as the starting point for the development of a new antibiotic.

## Figures and Tables

**Table 1:** Genome features

	*Chromobacterium sp.* HL1	*Alcaligenes parafaecalis* HL2
Genome length (bp)	5 226 257	4 056 399
Number of contigs	1	1
Largest contig (bp)	5 226 257	4 056 399
GC content (%)	64.4	56.1
N50 (bp)	5 226 257	4 056 399
Number of CDSs	4 591	3 649
Number of rRNAs	25	9
Number of tRNAs	84	57
Completeness (%)	99.15	100
Contamination (%)	0.85	0

**Table 2:** eggNOG categories of coding proteins

Class	Description	*Chromobacterium sp.* HL1 [%]	*Alcaligenes parafaecalis* HL2 [%]
**Information storage and processing**			
J	Translation, ribosomal structure, and biogenesis	186 [5.04]	190 [6.15]
A	RNA processing and modification	0	1 [0.03]
K	Transcription	334 [9.05]	353 [11.43]
L	Replication, recombination, and repair	129 [3.49]	101 [3.27]
B	Chromatin structure and dynamics	0	1 [0.03]
**Cellular processes and signaling**			
D	Cell cycle control, cell division, chromosome partitioning	43 [1.16]	43 [1.39]
Y	Nuclear structure	0	0
V	Defense mechanisms	49 [1.33]	42 [1.36]
T	Signal transduction mechanisms	168 [4.55]	77 [2.49]
M	Cell wall/membrane/envelope biogenesis	264 [7.15]	167 [5.41]
N	Cell motility	96 [2.60]	39 [1.26]
Z	Cytoskeleton	0	0
W	Extracellular structures	0	0
U	Intracellular trafficking, secretion, and vesicular transport	62 [1.68]	91 [2.95]
O	Post-translational modification, protein turnover, chaperones	117 [3.17]	98 [3.17]
**Metabolism**			
C	Energy production and conversion	257 [6.96]	242 [7.83]
G	Carbohydrate transport and metabolism	127 [3.44]	114 [3.69]
E	Amino acid transport and metabolism	292 [7.91]	247 [8.00]
F	Nucleotide transport and metabolism	96 [2.60]	104 [3.37]
H	Coenzyme transport and metabolism	191 [5.17]	132 [4.27]
I	Lipid transport and metabolism	107 [2.90]	117 [3.79]
P	Inorganic ion transport and metabolism	246 [6.66]	245 [7.93]
Q	Secondary metabolites biosynthesis, transport, and catabolism	91 [2.47]	102 [3.30]
**Poorly characterized**			
R	General function prediction only	0	0
S	Function unknown	836 [22.65]	583 [18.87]
**All proteins**		3691	3089

**Table 3:** BGCs identified with antiSMASH in the analyzed genomes

Sample name	*Chromobacterium sp.* HL1		Sample name	*Alcaligenes parafaecalis* HL2
# of BGC	13		# of BGC	7
Betalactone	1		Betalactone	2
Hserlactone	1		Ectoine	1
Hydrogen-cyanide	1		Hydrogen-cyanide	1
Isocyanide	1		NRPS	1
NRPS	6		T1PKS	1
RiPP-like	2		Terpene	1
Terpene	1			

**Notes: 1) NRPS includes different types of NRPS. 2) If multiple BGC types listed, the first type was chosen unless NRPS was listed, which is included in NRPS. 3) The two betalactone BGCs may be different for *Alcaligenes parafaecalis* HL2.

## References

[1] Aloke C, Achilonu I (2023). Coping with the ESKAPE pathogens: Evolving strategies, challenges and future prospects. Microb Pathog.

[2] Reddy BVB, Kallifidas D, Kim JH, Charlop-Powers Z, Feng Z, Brady SF (2012). Natural Product Biosynthetic Gene Diversity in Geographically Distinct Soil Microbiomes. Appl Environ Microbiol.

[3] Cheung GYC, Bae JS, Otto M (2021). Pathogenicity and virulence of *Staphylococcus aureus*. Virulence. 12(1):547-569. doi:10.1080/21505594.

[4] Kourtis AP, Hatfield K, Baggs J (2019). Vital Signs: Epidemiology and Recent Trends in Methicillin-Resistant and in Methicillin-Susceptible *Staphylococcus aureus* Bloodstream Infections — United States. Morb Mortal Wkly Rep.

[5] Taylor TA, Unakal CG (2024). *Staphylococcus aureus* Infection. In: StatPearls. StatPearls Publishing; 2024. Accessed May 30.

[6] Okwu MU, Olley M, Akpoka AO, Izevbuwa OE (2019). Methicillin-resistant *Staphylococcus aureus* (MRSA) and anti-MRSA activities of extracts of some medicinal plants: A brief review. AIMS Microbiol.

[7] Kapley A, Tanksale H, Sagarkar S (2016). Antimicrobial activity of *Alcaligenes sp*. HPC 1271 against multidrug resistant bacteria. Funct Integr Genomics.

[8] Pauer H, Hardoim CCP, Teixeira FL (2018). Impact of violacein from *Chromobacterium violaceum* on the mammalian gut microbiome. PLOS ONE.

[9] Wick R (2024). rrwick/Filtlong. Published online July 19, 2024. Accessed July 22.

[10] Li H (2016). Minimap and miniasm: fast mapping and de novo assembly for noisy long sequences. Bioinformatics.

[11] Kolmogorov M, Yuan J, Lin Y, Pevzner PA (2019). Assembly of long, error-prone reads using repeat graphs. Nat Biotechnol.

[12] nanoporetech/medaka (2024). Published online July 15, 2024. Accessed July 22.

[13] Wick RR, Schultz MB, Zobel J, Holt KE (2015). Bandage: interactive visualization of de novo genome assemblies. Bioinformatics.

[14] Parks DH, Imelfort M, Skennerton CT, Hugenholtz P, Tyson GW (2015). CheckM: assessing the quality of microbial genomes recovered from isolates, single cells, and metagenomes. Genome Res.

[15] Wick R (2024). rrwick/Polypolish. Published online July 21, 2024. Accessed July 22.

[16] Schwengers O, Jelonek L, Dieckmann MA, Beyvers S, Blom J, Goesmann A (2021). Bakta: rapid and standardized annotation of bacterial genomes via alignment-free sequence identification. Microb Genomics.

[17] Huerta-Cepas J, Szklarczyk D, Heller D (2019). eggNOG 5.0: a hierarchical, functionally and phylogenetically annotated orthology resource based on 5090 organisms and 2502 viruses. Nucleic Acids Res.

[18] Blin K, Shaw S, Kloosterman AM (2021). antiSMASH 6.0: improving cluster detection and comparison capabilities. Nucleic Acids Res.

[19] Ondov BD, Starrett GJ, Sappington A (2019). Mash Screen: high-throughput sequence containment estimation for genome discovery. Genome Biol.

[20] Brown CT, Irber L (2016). sourmash: a library for MinHash sketching of DNA. J Open Source Softw.

[21] Meier-Kolthoff JP, Göker M (2019). TYGS is an automated high-throughput platform for state-of-the-art genome-based taxonomy. Nat Commun.

[22] Ciccarelli FD, Doerks T, von Mering C, Creevey CJ, Snel B, Bork P (2006). Toward automatic reconstruction of a highly resolved tree of life. Science.

[23] De Pascale G, Nazi I, Harrison PHM, Wright GD (2011). β-Lactone natural products and derivatives inactivate homoserine transacetylase, a target for antimicrobial agents. J Antibiot (Tokyo).

[24] Mahizan NA, Yang SK, Moo CL (2019). Terpene Derivatives as a Potential Agent against Antimicrobial Resistance (AMR) Pathogens. Molecules.

[25] Raffa N, Won TH, Sukowaty A (2021). Dual-purpose isocyanides produced by *Aspergillus fumigatus* contribute to cellular copper sufficiency and exhibit antimicrobial activity. Proc Natl Acad Sci.

[26] Gomes ES, Schuch V, de Macedo Lemos EG (2014). Biotechnology of polyketides: New breath of life for the novel antibiotic genetic pathways discovery through metagenomics. Braz J Microbiol.

